# Amino Acids as Metabokines in Hypercatabolic States: Rethinking Nutritional Protein-Based Strategies Beyond Caloric Support

**DOI:** 10.3390/nu18111703

**Published:** 2026-05-27

**Authors:** Giovanni Corsetti, Evasio Pasini

**Affiliations:** 1 Department of Clinical and Experimental Sciences, Università di Brescia, 25128 Brescia, Italy; 2 Institute of Human Health, Ruaha Catholic University, Iringa P.O. Box 774, Tanzania; evpasini@gmail.com

**Keywords:** hypercatabolism, sarcopenia, cachexia, amino-acids, protein metabolism, critical illness

## Abstract

The clinical management of nutrition in acute and chronic diseases requires an integrated understanding of the interactions between energy intake, dietary protein, and amino acids (AAs). Many conditions (including sepsis, major trauma, cancer cachexia, chronic heart failure, chronic obstructive pulmonary disease, renal and liver failure, autoimmune diseases, and aging) share a common pathophysiological feature: the hypercatabolic state (HCS). HCS is characterized by systemic inflammation and neuroendocrine activation that increase basal metabolic rate, induce insulin resistance, and accelerate skeletal muscle proteolysis, leading to negative nitrogen balance, sarcopenia, and cachexia. Under these conditions, skeletal muscle acts as a metabolic reservoir of AAs mobilized to support energy production, gluconeogenesis, immune function, and vital organ metabolism, often at the expense of lean body mass and clinical outcomes. This narrative review examines the distinct and non-overlapping roles of calories, proteins, and AAs in metabolic regulation, with a particular focus on HCS. Calories primarily act as a permissive factor for protein utilization, whereas proteins and especially essential amino acids (EAAs) function not only as substrates for protein synthesis but also as signaling molecules (metabokines) regulating anabolic and catabolic pathways, including mTORC1 and AMPK. Energy provision alone is insufficient to prevent muscle loss when EAA availability is inadequate, while high protein intake without sufficient energy fails to sustain anabolism due to anabolic resistance. Evidence indicates that protein quality and the balanced availability of all EAAs are more critical for lean mass preservation than total caloric intake alone. Strategies based solely on calorie provision or protein quantity are therefore limited, whereas targeted EAA supplementation may partially overcome anabolic resistance in selected hypercatabolic conditions. Overall, this review supports a shift from calorie-centered nutrition toward a signal-based, quality-oriented approach, based on personalized needs, that integrates metabolic status, protein quality, and AA signaling to preserve lean body mass and improve clinical outcomes.

## 1. Introduction

The central clinical problem in the interaction between energy (calories), proteins, and amino acids (AAs) is the need to integrate physiological and metabolic knowledge to optimize nutritional therapy, adapting it to the patient’s physio-pathological conditions and their specific energy and protein needs [1]. Despite their clinical diversity, acute and chronic diseases such as sepsis, severe trauma, advanced cancer with cachexia, chronic heart failure, COPD, renal and liver failure and/or autoimmune diseases share a unifying physio-pathological metabolic denominator: the hypercatabolic state (HCS) [2]. It is defined as a pathological state where systemic inflammation and neuroendocrine activation drive the basal metabolic rate far beyond physiological necessity, creating a devastating energy gap and a massive shift in protein metabolism. Indeed, chronic inflammation interacts bidirectionally with metabolism through hormonal and neural signals of the neuroendocrine axis, particularly the hypothalamic–pituitary–adrenal axis, thereby shaping systemic energy homeostasis and disease risk [3].

To meet the increased energy demands and the need for biosynthetic precursors, the body initiates a significant process of muscle proteolysis induced by a potent cocktail of pro-inflammatory cytokines and stress hormones, which simultaneously promote catabolism and induce anabolic resistance, locking the patient in a cycle of self-consumption, particularly of the skeletal muscle [4].

In the HCS, skeletal muscle is no longer viewed as a motor for movement, but as a primary reservoir of “totipotent” biochemical molecules: AAs. Once liberated through breakdown, these AAs undergo deamination and their carbon skeletons are strategically repurposed as versatile metabolic intermediates. They can be funneled into the Krebs cycle to sustain ATP production or used as substrates for gluconeogenesis and lipogenesis, supporting the high-intensity metabolic requirements of the immune system and vital organs.

Clinically, this aspect is of extreme importance because this systematic “mining” of AAs predominantly from muscle tissue, if not identified and treated with specific therapeutic strategies, causes loss of lean body mass in patients, leading to the development of sarcopenia and subsequently cachexia. These independent factors can influence the patient’s quality of life, the length of hospital stay and/or recovery time, and even their survival to a much greater extent than total caloric intake [5,6,7,8]. Therefore, in the HCS, it is important to understand how total caloric balance modulates protein utilization and tissue preservation. It is crucial to distinguish between “calories” as units of energy and “proteins” as functional biological molecules. We believe that understanding these biochemical mechanisms is crucial for the management of protein/caloric malnutrition in HCS patients.

Significant areas of uncertainty remain in the relationship between AAs and HCS, especially at the mechanistic and clinical levels. It is still incompletely understood how, when, and in what proportions AAs, particularly EAAs, can overcome anabolic resistance and effectively counteract protein loss in the HCS [9,10].

Although there are many recommendations aimed at defining the optimal protein intake, the individual nature of the metabolic response makes it difficult to standardize dosages for different clinical populations [11]. While proteins can act as energy substrates, not all of the AAs derived from them are metabolized in the same way; instead, they may have different metabolic fates based on their biochemical characteristics. Beyond the traditional classification into essential and non-essential, AAs should not be viewed merely as passive substrates for protein synthesis or energy production. Instead, each AA possesses unique biochemical properties that allow it to function as a distinct signaling molecule. These individual AAs can trigger specific metabolic pathways and modulate cellular responses. Due to this regulatory capacity, several AAs have been increasingly recognized as “metabokines” demonstrating a sophisticated ability to orchestrate systemic metabolism far beyond their structural roles [12,13].

While calories represent a general energy concept, proteins constitute biological structures and catalysts (enzymes) without which cellular functions are not possible. Furthermore, protein metabolism involves energetic costs and metabolic responses that are not adequately described by calorie counting alone. This distinction is important when evaluating nutrition, metabolism, energy balance, and clinical outcomes [1,14]. As a consequence, a nutritional approach based solely on calories has obvious limitations: (i) it ignores individual variability in metabolism, (ii) it fails to consider the qualitative effects of nutrients, (iii) it neglects metabolic and psychological adaptations, and (iv) it may be insufficient to explain the complex relationships between diet, body composition, and metabolic health.

Recent literature supports a more nuanced paradigm that combines energy quantity with nutritional quality, individual physiology, and dynamic metabolic processes [15,16,17]. The importance of the close interdependence between energy, dietary protein sources, and AA composition in regulating metabolism is a fundamental concept that must guide different nutritional strategies across different physiological and pathological conditions [4].

This narrative review aims to provide an integrated view of the evidence on how calories, proteins, and AAs have profoundly different effects on metabolism, especially in the HCS conditions. These elements interact to determine dietary quality and energy balance, and they regulate biological pathways to influence metabolic health.

This paper is therefore designed to rethink and challenge existing nutritional paradigms, rather than present a systematic review or introduce new findings, encouraging the reader to consider broader conceptual frameworks in nutrition.

## 2. Methods

We considered the relevant scientific literature in databases as PubMed, Scopus, WOS and Embase, focusing on the most recent studies (generally from the last ten years), using a combination of keywords such as “protein metabolism”, “essential amino acids”, “hypercatabolism”, “energy overfeeding” and “critical illnesses”. Priority was given to basic and clinical research studies. Additionally, meta-analyses, systematic reviews, and narrative reviews were considered. To improve the transparency of all aspects of this narrative and qualitative research, we followed the guidelines proposed by the Standards for Reporting Qualitative Research (SRQR) [18].

## 3. Protein Metabolism: Beyond Energy Provision

It is necessary to separate the concept of protein from that of an “energy substrate”.

In terms of energy, protein catabolism provides an average of about 4 kcal per gram. However, unlike carbohydrates and lipids, proteins are not a “pure” energy substrate. In fact, part of their potential energy is lost during the digestion of dietary proteins and throughout AA metabolism, including protein synthesis, deamination and transamination reactions, as well as the synthesis and excretion of urea. Consequently, the net energy yield from proteins is relatively low. The primary function of proteins (as their name implies) is structural and functional rather than energy-producing.

Under physiological conditions, when the body is not in the HCS, energy balance is stable, and the digestive system is functioning properly, ingested proteins are broken down into their fundamental building blocks: AAs. Once absorbed into the bloodstream, these AAs are mainly utilized for the synthesis of new proteins that are essential for life. This complex assembly process is highly regulated and far from random; it is finely tuned to metabolic demands and controlled by specific molecular signaling pathways.

Given this mechanism, the quality and digestibility of dietary proteins become paramount. “High-quality” proteins are defined as those that provide an abundant supply of essential AAs (EAAs), which the human body cannot synthesize endogenously. However, even the best dietary protein sources rarely contain more than approximately 40% EAAs. Despite this limitation, their presence is crucial because EAAs do far more than serve as mere building blocks, as they also act as key regulators of the metabolic pathways that initiate and sustain new protein synthesis [19,20]. Recent literature suggests that the concept of protein metabolism extends beyond traditional measures of intake, incorporating aspects such as food source, protein quality, and interactions with metabolic signaling pathways [21]. Some of the most recent reviews of protein metabolism are summarized in Table 1.

Understanding protein quality through metrics such as the digestible indispensable amino acid score (DIAAS) deepens our understanding of the metabolic impacts of dietary protein, linking digestibility and EAA content with anabolic responses [26]. Furthermore, protein/AAs-induced modulation of insulin resistance aligns metabolic regulation with anabolic signaling pathways, highlighting the complex interactions between diet and metabolism [24]. Consequently, adequate daily protein intake supplies the AAs necessary for body protein synthesis and essential metabolic functions. Indeed, protein metabolism is very complex and regulated by sensing and signaling networks involving hormones, regulatory molecules, and multiple upstream and downstream pathways [27].

To emphasize the multifactorial role of proteins, their main functions beyond energy provision are summarized as follows.

Proteins act as structural (plastic) substrates, providing cells with a dynamic and adaptable architecture. Their ability to undergo conformational changes, post-translational modifications, and regulated assembly into higher-order complexes enables cellular remodeling in response to mechanical, biochemical, and environmental stimuli, thereby supporting adaptability and homeostasis [28,29,30,31]. In addition, food proteins contain peptide sequences capable of exerting numerous physiological effects, such as antioxidant, antihypertensive, immunomodulatory, and anti-inflammatory activities [32,33]. Indeed, proteins also function as central mediators of metabolic signaling, integrating nutrient availability with cellular and systemic responses. Protein hormones such as insulin regulate anabolic pathways via receptor-mediated signaling [34], while intracellular protein complexes such as mTORC1 act as nutrient and energy sensors controlling protein synthesis, autophagy, and mitochondrial metabolism [35,36]. AAs, particularly leucine, directly modulate mechanistic target of rapamycin complex-1 (mTORC1) activity through specific protein sensors [37,38], whereas energy stress activates AMPK to promote catabolic pathways and inhibit anabolic signaling [39]. In parallel, reversible post-translational modifications further fine-tune metabolic regulation [40]. Secreted proteins such as myokines, adipokines, and hepatokines coordinate whole-body energy homeostasis, with FGF21 representing a key example of endocrine metabolic regulation [41]. Finally, proteins serve as immune modulators, orchestrating both innate and adaptive immune responses. Cytokines and chemokines regulate immune cell activation through protein-mediated signaling pathways [42,43], while pattern recognition receptors such as Toll-like receptors (TLRs) initiate host defense mechanisms upon pathogen detection [44]. Immune checkpoint proteins, including PD-1 and CTLA-4, ensure immune tolerance by negatively regulating T-cell activation [45]. Recent evidence also highlights the immunomodulatory potential of specific protein–protein interactions, such as HSP60-derived peptides targeting TLR4/MD-2 complexes, underscoring their relevance for peptide-based immunotherapies [46,47].

In summary, food proteins and the bioactive peptides derived from them also play an important role in immune modulation, acting on finely regulated metabolic signaling pathways to maintain tissue and whole-organism homeostasis.

## 4. Fate of Proteins in Caloric Deficit

Under a caloric deficit, such as the HCS, the body’s protein metabolism undergoes significant adaptations to maintain essential physiological functions while simultaneously meeting energy needs. Energy deficiency shifts substrate utilization toward increased lipid oxidation. However, without adequate dietary protein intake coupled with resistance exercise, endogenous proteins, especially those from skeletal muscle, become a significant source of amino acids for gluconeogenesis and energy production [48]. This leads to a negative NB, characterized by elevated muscle breakdown, suppression of protein synthesis and consequent sarcopenia which, if not prevented, can become muscle cachexia with important consequences on the patient’s health [49].

Energy deficit triggers a complex hormonal cascade that promotes proteolysis to provide AAs for energy (gluconeogenesis). This catabolic state is caused by a reduction in anabolic hormones (insulin, testosterone) and an increase in catabolic hormones (cortisol, ghrelin) [15]. However, a randomized controlled trial of 39 adults assigned to diets providing daily protein at 0.8 (RDA), 1.6, and 2.4 g/kg for 31 days, showed that even under hypocaloric conditions, increased protein intake can attenuate lean mass loss by stimulating muscle protein synthesis and reducing proteolysis. This suggests that consuming dietary protein at levels exceeding the RDA may protect muscle mass during short-term weight loss [50,51]. Consistent with this observation, a study conducted on muscle biopsies from 15 healthy but overweight men subjected to calorie restriction (3.2 kcal/kg body weight/day) plus exercise (45 min of one-arm cycling + 8 h of walking) for 4 days, followed by a control diet for 3 days, with a caloric content similar to the pre-intervention diet, demonstrated that in cases of severe energy deficit, levels of phosphorylate glycogen synthase kinase-3β (pSer9GSK3β) decrease and skeletal muscle becomes refractory to the anabolic effects of whey protein ingestion, regardless of contractile activity. These muscle changes were associated with changes in leptin, insulin, AAs, cortisol, total cortisol/testosterone, and lean mass [52]. Another study involving 28 male college students not performing resistance training showed that a high protein intake alone was insufficient to prevent lean mass loss associated with a 6-week moderate energy restriction [53]. More recent literature also suggests that protein quality, AA composition, and meal timing play a critical role in determining protein fate during caloric deficits, with important implications for metabolic health, immune competence, and physical performance [54].

### Protein Sparing Effect of Calories

Under normal conditions, the human body uses glucose and fat as its main energy sources. Only when energy intake is insufficient is the body forced to mobilize muscle protein reserves to convert them into glucose (gluconeogenesis) for energy, accelerating protein loss and compromising lean mass [55]. In fact, historical metabolic studies conducted on pigs demonstrated that the introduction of carbohydrates into low-protein or low-calorie diets can significantly improve nitrogen retention, highlighting a marked protein-sparing effect [56]. Therefore, adequate calorie intake reduces the need for protein breakdown and protects against muscle catabolism. This effect has important clinical implications, as inadequate caloric intake, even in the presence of sufficient protein intake, can lead to an increase in protein turnover and a loss of lean mass.

The protein-sparing effect of calories describes the phenomenon whereby adequate energy intake, primarily in the form of carbohydrates and/or lipids, reduces the degradation of endogenous proteins (primarily muscle), thus preserving lean mass in conditions of caloric deficit or catabolism. This effect has important clinical implications, as inadequate caloric intake, even in the presence of sufficient protein intake, can lead to an increase in protein turnover and a loss of lean mass. This concept underpins many clinical nutritional strategies, such as the protein-sparing modified fast (PSMF) and nutritional support therapies in critically ill patients [57,58,59,60]. PSMF therapies have been developed to address protein-energy malnutrition in hospitalized patients, particularly those with metabolic stress (e.g., trauma, sepsis, etc.), demonstrating a reduction of nitrogen loss and preservation of lean mass [58].

We must emphasize that the HCS are characterized by increased energy expenditure, systemic inflammation, insulin resistance, and accelerated muscle protein degradation. In this context, maintaining lean body mass depends critically on the interaction between total caloric intake and protein intake, rather than the amount of protein or calories administered alone. In severe acute HCS conditions, even a high protein intake may be insufficient to counteract muscle catabolism, because the body develops anabolic resistance to AAs [61,62].

This metabolic feature is confirmed by a clinical trial which shows that high-dose protein intake in the acute phases of 16,000 critically ill patients fails to demonstrate an improvement in mortality, indicating that the energy-protein balance is complex and not always linear [61]. Understanding the metabolic mechanisms underlying the protein-sparing effect represents a key element in planning nutritional interventions aimed at preserving muscle mass and improving clinical outcomes.

## 5. Protein Turnover and Nitrogen Balance

Protein turnover is a critical process for maintaining tissue homeostasis and metabolic health. Nitrogen balance (NB) is a classic indicator of body protein status, reflecting the difference between the amount of nitrogen consumed and excreted; this correlates with net protein gain or loss [63]. A positive NB indicates anabolic states, while a negative NB is characteristic of catabolic conditions such as fasting, disease, aging, and energy deficit [64]. Protein turnover is highly dynamic and regulated by dietary protein intake, AA availability, hormonal signals (e.g., insulin, glucocorticoids), and physiological stressors (e.g., exercise or inflammation) [65,66]. However, while the NB provides valuable integrative information at the whole-body level, it is not sensitive enough to detect tissue-specific changes in protein metabolism, particularly in skeletal muscle [67,68].

A systematic review and meta-analysis of 395 individuals estimated a mean nitrogen requirement of 104.2 mg N/kg/day. No significant differences were found by sex, age (<60 vs. ≥60 years), climate, or protein source (animal, plant, or mixed). Despite consistency with previously reported values, substantial inter-study heterogeneity limits the strength of the conclusions [69].

In a balanced, randomized, double-blind crossover study involving 14 young, healthy, moderately to well-trained participants, metabolic and hormonal responses to isocaloric whey protein (1.2 g/kg) versus carbohydrate intake were compared. Protein intake stimulated insulin secretion independently of glucose, mediated by increased plasma amino acids and GLP-1, whereas glucose intake elicited higher GIP levels. Protein-only intake also led to increased urinary nitrogen excretion, mainly within 2–8 h and persisting up to 24 h [70].

A recent meta-analysis quantitatively compared protein requirements derived from the NB method (777 participants) with those obtained using the indicator AA oxidation method (IAAO; 186 participants). The protein requirements estimated by the IAAO were approximately 30% higher than those calculated using NB [71]. These findings suggest a potential paradigm shift in the assessment of protein requirements in humans and underscore the need to re-evaluate current recommendations across different physiological conditions.

## 6. More than Just Building Blocks: Essential, Non-Essential and Conditionally Essential Amino Acids

A common misconception in clinical nutrition is the tendency to regard dietary protein as a monolithic entity, often detached from the specific AAs that compose it. The nutritional and metabolic value of a protein is determined by its AA composition, as each individual AA possesses distinct biosynthetic origins and autonomous metabolic functions. In human nutrition, proteinogenic AAs are traditionally classified into three major categories based on the body’s capacity to synthesize them and their dietary necessity.

EAAs are those that the human body cannot synthesize endogenously in sufficient quantities to meet physiological demands and therefore must be supplied through the diet. Humans require nine EAAs (histidine, isoleucine, leucine, lysine, methionine, phenylalanine, threonine, tryptophan, and valine). A deficiency in any of these AAs leads to impaired protein synthesis and adverse effects on growth, maintenance, and metabolic homeostasis because they cannot be produced quickly enough from other substrates under normal conditions [14,72]. It follows that supplementation with one EAA or a few EAAs, such as only BCAAs, could lead to effects opposite to those expected. In fact, a deficiency of other EAAs can promote muscle protein breakdown to obtain the deficient EAAs.

By contrast, NEAAs (alanine, asparagine, aspartic acid, glutamic acid, glutamine, glycine, pProline, serine, cysteine, tyrosine) are those that the body can synthesize de novo at rates sufficient to satisfy typical physiological requirements in healthy adults. Under basal conditions, these NEAAs do not require dietary intake because endogenous metabolic pathways generate them from intermediary metabolites such as glycolytic or tricarboxylic acid (TCA) cycle intermediates [73].

The distinction between EAAs and NEAAs is not absolute but depends on the physiological context and metabolic demand. This leads to the classification of certain AAs as conditionally essential.

Conditionally essential or semi-essential (CEAAs), such as glutamine, cysteine, tyrosine, glycine andoProline, are typically considered NEAAs under normal, healthy conditions because the body can synthesize them. During periods of rapid growth, physiological stress, illness, trauma, or specific life stages (e.g., infancy or pregnancy), the rate of endogenous synthesis may become insufficient relative to metabolic demand, rendering their exogenous supply necessary. Common examples include arginine, cysteine, glutamine, glycine, proline, and tyrosine [14]. This conditional essentiality reflects a dynamic nutritional requirement: for example, arginine synthesis may not meet demand in young children or individuals with severe catabolic stress, and glutamine demand can exceed endogenous production during critical illness or injury. Metabolic stressors can turn normally NEAAs into nutritionally limiting substrates, with implications for clinical nutrition and dietary planning [14]. From a nutritional standpoint, the categorization of AAs into these three groups has important implications for protein quality assessment and dietary recommendations, including the evaluation of limiting AAs in dietary proteins and the formulation of clinical nutrition strategies for vulnerable populations [73].

In summary, while calories fulfill energetic requirements, AAs, particularly EAAs, represent the limiting factor for protein synthesis, especially in the HCS conditions.

## 7. Hypercatabolic States and Nutritional Implications

Hypercatabolism is a complex metabolic condition driven by systemic inflammation and stress responses, in which catabolic processes overwhelm anabolic pathways, leading to increased energy expenditure and accelerated loss of body protein. Pro-inflammatory cytokines (TNFα, IL-1, IL-6) and stress hormones (cortisol, catecholamines, and glucagon) induce insulin resistance, enhance proteolysis and gluconeogenesis, and suppress anabolic signaling. In addition, mitochondrial dysfunction further reduces metabolic efficiency. These mechanisms result in muscle wasting, impaired immune function, and worsened clinical outcomes, particularly in critical illness and severe trauma [5,7,48,74].

### 7.1. Mechanistic Basis of the Hypercatabolic State

An HCS state is characterized by a sustained imbalance between anabolic and catabolic processes, resulting in net tissue breakdown, particularly skeletal muscle proteolysis, despite adequate or even elevated energy intake. This condition is commonly observed in aging, critical illness, cancer cachexia, chronic inflammation, prolonged inactivity, and severe metabolic stress. At the molecular level, hypercatabolism arises from the dysregulation of key nutrient and energy-sensing pathways, most notably mTOR, AMPK, and the ubiquitin–proteasome system (UPS).

The mechanistic target of rapamycin complex 1 (mTORC1) is the central anabolic hub that integrates signals from EAA (particularly leucine), insulin/IGF-1, mechanical loading, and cellular energy status to stimulate protein synthesis and inhibit autophagy. In the HCS, mTORC1 activity is markedly reduced due to multiple converging factors, including AA insufficiency, insulin resistance, inflammatory cytokines, and energetic stress [36]. Reduced mTORC1 signaling leads to diminished phosphorylation of downstream effectors such as S6 kinase 1 (S6K1) and 4E-BP1, resulting in impaired translation initiation and reduced muscle protein synthesis [75]. Importantly, even when caloric intake is preserved, inadequate essential amino acid signaling—especially leucine—limits mTOR activation, highlighting that energy availability alone is insufficient to maintain an anabolic state [76].

Hypercatabolism is also associated with chronic activation of AMP-activated protein kinase (AMPK), a master sensor of cellular energy stress activated by increased AMP/ATP and ADP/ATP ratios. AMPK promotes catabolic pathways to restore energy homeostasis while actively suppressing anabolic processes, including protein synthesis, lipid synthesis, and cell growth [39]. AMPK inhibits mTORC1 both directly, through phosphorylation of raptor, and indirectly, via activation of the TSC1/TSC2 complex, further reinforcing anabolic resistance [77]. In skeletal muscle, persistent AMPK activation shifts metabolism toward fatty acid oxidation and mitochondrial biogenesis but simultaneously exacerbates muscle wasting when not counterbalanced by sufficient anabolic signaling [78].

The UPS represents the primary pathway for selective protein degradation in hypercatabolic conditions. Activation of the UPS is driven by stress hormones (glucocorticoids), inflammatory mediators (TNF-α, IL-6), oxidative stress, and reduced insulin/IGF-1 signaling [79]. At the transcriptional level, catabolic states induce forkhead box O (FoxO) transcription factors, which upregulate muscle-specific E3 ubiquitin ligases such as MuRF1 (muscle RING finger-1) and Atrogin-1/MAFbx. These ligases tag myofibrillar proteins for degradation by the 26S proteasome, leading to rapid loss of muscle mass and function [80]. Notably, FoxO activation is normally suppressed by Akt signaling downstream of insulin and IGF-1. In hypercatabolism, insulin resistance and inflammatory signaling blunt Akt activity, releasing FoxO-mediated transcription of proteolytic genes and amplifying muscle breakdown [81].

Therefore, the HCS reflects a systems-level failure of anabolic signaling, in which: mTORC1 activity is suppressed due to inadequate AA signaling and hormonal resistance, AMPK remains chronically activated, reinforcing energy-conserving and anti-anabolic programs; and the UPS is upregulated, driving accelerated proteolysis.

Crucially, this condition demonstrates that catabolism is not merely a consequence of a caloric deficit, but rather the result of impaired nutrient signaling, endocrine dysfunction, and inflammatory stress. This mechanistic framework supports a signal-based nutritional and therapeutic approach, emphasizing essential amino acid availability, anabolic sensitivity, and modulation of metabolic signaling pathways rather than caloric intake alone.

### 7.2. Nutritional Implications

Nutritional status is a key determinant across all HCS conditions, from physiological aging to cancer. Malnutrition is highly prevalent in older adults, affecting ~45% of community-dwelling individuals, over 50% of hospitalized patients, and up to 84–100% of those in long-term care [82]. These conditions adversely impact clinical outcomes and prognosis. In oncology, 30–90% of patients are malnourished due to reduced intake, metabolic and digestive alterations, and treatment-related effects. Moreover, persistent HCS in cancer drives systemic protein catabolism, leading to sarcopenia and, if untreated, progression to cachexia, a major contributor to mortality [83,84].

Impaired protein turnover is the most important metabolic consequence underlying all chronic HCS conditions. Specifically, the degradation of skeletal muscle and globular proteins, such as albumin, releases AAs to meet the body’s essential energy needs, thereby reducing skeletal and cardiac physiological and metabolic functions [5,74].

Studies on chronic stable heart failure patients show that about 30% of patients exhibit reduced serum albumin (<3.5 g/dL) [85], and these conditions are related to increased morbidity, hospitalization, and mortality, independent of the primary diseases [86,87]. Eating-related protein disorders are common in most chronic HCS patients. Indeed, up to 50% of patients with severe chronic disease exhibit altered protein metabolism, contributing to inadequate nutritional intake and reduced availability of all nutrients, particularly EAAs. Protein disarray also affects proteins that regulate and/or guarantee biochemical functions and/or structure of various body organs and/or systems [74].

Adequate caloric intake alone is insufficient to prevent skeletal muscle loss in the absence of EAAs, which are essential substrates and signaling regulators of muscle protein synthesis (MPS), notably via mTORC1 [35]. During energy restriction, increased calories from non-nitrogenous sources fail to sustain post-exercise MPS unless sufficient EAAs are provided; accordingly, only EAA-enriched proteins, not carbohydrates, preserve post-exercise anabolism during starvation [88]. Consistently, in older humans, low doses of EAAs significantly stimulate muscle fractional synthetic rate independent of total caloric intake [89], and EAA supplementation (8 g/kg/day) during hypocaloric diets preferentially preserves lean mass over fat mass, highlighting that calories alone cannot prevent muscle catabolism without adequate EAAs [90].

During aging and conditions of increased metabolic demand, inadequate EAA and energy intake can compromise health and lifespan. Preclinical evidence from isocaloric, iso-nitrogenic diets with varying EAA/NEAA ratios shows an inverse relationship between lifespan and dietary NEAA content. Both EAA restriction and NEAA excess induced rapid, irreversible alterations in muscle and adipose tissue, independent of caloric intake. Even with increased calories, EAA deficiency led to wasting and reduced longevity, highlighting balanced EAA intake, via high-quality protein or supplementation, as a key strategy for health preservation [72].

Dietary protein can exert a true lean mass maintenance effect only when supported by an adequate total caloric intake as recently demonstrated. A single-center, open-label, randomized, controlled clinical trial, 220 acutely ill adult hospitalized patients at nutritional risk (NRS-2002 ≥ 3), but without severe hypophagia, and with an expected length of stay ≥ 7 days, were randomly assigned to receive a high-protein oral nutritional supplement (containing 300 kcal and 18 g of protein, twice a day) or nutritional supplement on demand. At discharge, patients receiving nutritional supplementation improved muscle mass evaluated by bioelectrical impedance analysis (BIA, phase angle mean difference = 0.49 [95%CI, 0.33 to 0.64], *p* < 0.001), body weight, and muscle handgrip strength (kg, mean difference = 0.8 [0.1, 1.5], *p* < 0.042). Additionally, hospital stay was reduced by two days [91].

In the HCS, energy intake acts as a permissive factor enabling proteins and AAs to exert their structural, functional, and protein-sparing roles; however, increased protein intake fails to preserve lean mass in the absence of adequate energy, while sufficient calories without EAAs promote muscle loss. Overall, the anabolic response to nutritional support is primarily constrained by EAA availability rather than caloric content alone. Moreover, in the HCS, energy provision per se does not suppress muscle protein breakdown, as excess calories are preferentially oxidized or stored as fat without stimulating muscle protein synthesis. Even with optimal caloric and protein intake, severe HCS is characterized by persistent anabolic resistance driven by chronic inflammation and insulin resistance [92], limiting the effective availability of EAAs from dietary protein. Thus, supplementation with all EAAs in balanced ratios is required to overcome this metabolic constraint and mitigate HCS.

Consistent with this evidence, dietary protein, free-form AAs, and energy intake play distinct and non-overlapping roles. In the HCS with elevated muscle proteolysis, caloric intake alone is insufficient to preserve body protein stores; its efficacy depends on the availability of high–biological value protein and/or EAA supplementation. Inadequate EAA supply may render increased energy intake ineffective or even detrimental, potentially accelerating rather than preventing muscle protein loss. Table 2 summarizes the effect of total caloric intake combined with protein intake on the maintenance of lean mass in the HCS.

**Figure 1:**
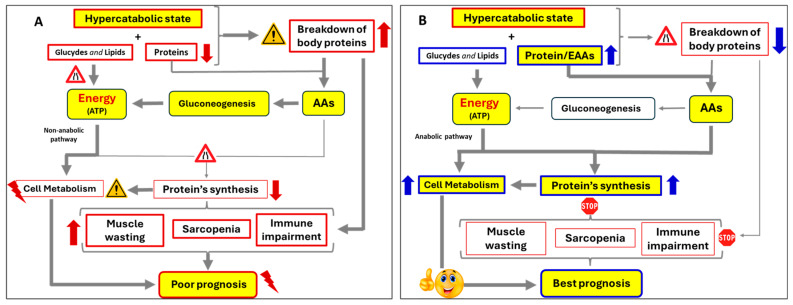
Schematic representation of the interaction between hypercatabolic states and protein/energy availability. (**A**) In the presence of a hypercatabolic state combined with inadequate protein and caloric intake, skeletal muscle proteolysis is enhanced to provide amino acids (AAs), particularly essential amino acids (EAAs). These substrates are predominantly redirected toward energy production (thick gray arrows) to support an already compromised cellular metabolism, rather than being utilized for anabolic processes. (**B**) When sufficient protein/EAAs and caloric intake are available, muscle proteolysis is attenuated. AAs and EAAs derived from dietary proteins and/or nutritional supplementation are preferentially used to sustain anabolic pathways (thick gray arrows), thereby counteracting the hypercatabolic state and mitigating its associated deleterious effects.

Figure 1 provides a schematic overview of the interaction between an HCS state and protein/energy availability and how this relationship influences prognosis.

## 8. Nutritional Supplementation of Proteins and EAA

In HCS, both whole protein supplementation and EAA supplementation (at least 8 g/day of balanced EAA mix) aim to counteract accelerated muscle protein breakdown and support anabolism, but they have distinct profiles of benefits and limitations. Whole protein sources (e.g., complete dietary proteins or protein powders) provide a broad spectrum of AAs that support overall nitrogen balance and have been associated with modest improvements in muscle mass, strength, and physical function in malnourished or elderly patients, although evidence quality varies and effects are sometimes small due to study heterogeneity and anabolic resistance in these populations (as shown in mixed clinical studies) [97,98]. Table 3 summarizes the main studies aimed at elucidating the relationship between energy and protein intake and the preservation of lean body mass (LBM) in HCS patients.

Excessive protein intake does not consistently exert a beneficial effect on LBM, as suggest recent systematic reviews of randomized controlled trials (RCTs) conducted in intensive care unit (ICU) patients (see Table 4).

We believe that the conflicting results emerging from studies in HCS patients with proteins supplementation could be due to the heterogeneity of the treatment protocols and evaluated outcomes.

Moreover, many factors capable of influencing these complex metabolic phenomena and, consequently, the clinical results were not adequately considered. Indeed, it is known that the biological quality of the proteins administered, total energy availability and the patient’s medical conditions could influence the magnitude and duration of postprandial muscle protein synthesis. Furthermore, this process is largely determined by protein’s content of EAAs and dietary protein digestion with subsequent AA intestinal absorption. All of them are essential factors capable of influencing AA blood availability and related muscle protein synthesis in humans [20]. These limitations highlight the need for further well-designed and comprehensive studies based on our complete knowledge of these complex metabolic and clinical phenomena.

Supplementation of a special mixture of free-form EAAs, which, therefore, does not have to be released by the digestion of food proteins but are readily assimilable and available in the blood of patients, is able to maintain muscle size and attenuate systemic inflammation in acute and subacute clinical settings, especially when enriched for key anabolic triggers such as leucine even in the context of reduced dietary intake [5,115,116]. However, if total protein intake is already sufficient, additional EAA alone may not confer further functional advantages and can be cost-inefficient (as highlighted in recent clinical nutrition analyses) [117]. A central practical difference is that protein supplementation also contributes to total caloric and nitrogen intake, which is valuable in generalized malnutrition, whereas isolated EAAs supplements bypass digestive protein processing and can be better tolerated in some patients with impaired digestion or appetite loss. It is important to note that an EAA supplement must contain all EAAs in balanced proportions to avoid incomplete anabolic signaling.

Preclinical studies demonstrate that the administration of specific balanced mixtures of EAAs exerts many beneficial effects on cell metabolism in different experimental conditions. It has been demonstrated that a diet with EAA deficiency significantly reduces survival in an EAA concentration-dependent manner, whereas EAA excess increases survival [118,119]. Under normal physiological conditions, the efficiency of recycling muscular proteins to obtain EAAs is limited (approximately 70%). As consequence, supplementation with a single EAA, even at higher doses, is not sufficient to maintain or increase muscle protein synthesis [68]. This effect is significantly worse in the HCS. This means that an anabolic state cannot be induced without the presence of all EAAs in adequate proportions required by the cellular metabolic state. It has been demonstrated that supplementation with a stoichiometrically balanced mixture of all free EAAs, formulated according to humans needs, promoted protein anabolism under numerous experimental conditions from senescence [120,121,122] to chemotherapy [123,124] and colon cancer cells in vitro and in vivo [125,126]. These effects depend on the activation of endothelial nitric oxide synthase (eNOS), which stimulates mitochondrial biogenesis in skeletal and cardiac muscle of mice and reduces the production of reactive oxygen species (ROS), as well as on the activation of mTORC1 [35,127]. From a translational point of view, this point is particularly important especially for patients with chronic diseases (such as diabetes, senescence and CHF), whose muscles have strongly compromised mitochondrial activities and heavy alterations in energy metabolism [128,129]. These observations indicate that specific individual AAs, much like vitamins, cytokines, or hormones, can influence key metabolic pathways. For this reason, they have been defined as metabokines [130]. Metabokines are small molecules (e.g., metabolic intermediates) that act as bioactive metabolites. In addition to their role in cellular metabolism, they act as signaling molecules, regulating biological functions similarly to cytokines. Specifically, they modulate the activation, differentiation, and function of immune cells, influencing inflammation, immune response, and tissue homeostasis [131].

The experimental results are confirmed in human beings. Healthy individuals who intake of 20–30 g of protein (or 0.25–0.30 g/kg body mass) or ~10 g of free-form EAAs (or 0.10 g/kg body mass) per dose optimally stimulate muscle protein synthesis particularly following resistance exercise and when energy availability is sufficient [132]. A study involving 16 healthy men and women demonstrated that a balanced EAA formulation combined with whey protein is highly anabolic compared with a whey protein–based recovery product. The anabolic response was approximately threefold and sixfold greater for the low and high doses of free EAA/protein, respectively, and was shown to be dose-dependent [133]. Another study performed on 38 healthy elderly subjects showed that an EAAs-based multi-ingredient nutritional supplementation of 12 weeks is not effective in changing the myoelectric manifestation of fatigue and time to perform the task failure, but can positively affect muscle mass, muscle strength, muscle power and VAT, counterbalancing more than one year of age-related loss of muscle mass and strength [134]. Interestingly, the increase in plasma concentration of free EAAs is more rapid than that of those derived from proteins in both young and elderly subjects. Free EAAs induce a greater anabolic effect than those derived from whole proteins due to the simultaneous arrival of all EAAs at the sites of protein synthesis [135].

Other clinical studies confirm the effects. Oral supplementation of special mixture of individual EAAs (45 g/day) added to normal protein intake in older subjects (>71 years) to 10 days bed rest, preserves muscle function during compulsory inactivity, suggesting that EAAs supplementation is potentially an efficient method of increasing protein intake without affecting satiety [136]. A stable isotope tracer study showed that a low dose (3.6 g) of a high-leucine EAAs formulation with arginine significantly increased muscle protein fractional synthetic rate in older adults, with ~80% of ingested EAAs incorporated into muscle protein. These findings indicate that low-dose EAAs formulations can effectively stimulate muscle protein synthesis in older adults [89].

Thus, consuming high-biological-value proteins (therefore with a high EAAs content) promotes protein metabolism throughout the body and, especially in older adults, in skeletal muscle. On the contrary, plant-based protein sources rich in fiber and micronutrients can be valuable, but they have less anabolic potential than animal proteins [137]. Notably, a mixture of free EAAs has positive effects also in patients with an HCS, such as heart failure. Several clinical studies show that the administration of balanced mixtures of free-EAAs can prevent malnutrition, enhance muscle mass and strength, and improve quality of life in these patients [74,115,116,138].

The effects of free EAAs are extensively studied in patients with sepsis-induced HCS. Septic patients have profound metabolic disturbances that impair protein synthesis with loss of skeletal muscle mass and strength, termed sepsis-associated muscle waste [139]. Sepsis prevents the increase in protein synthesis-induced by electrically stimulated muscle contraction by a mechanism likely dependent on mTOR [140]. EAAs supplementation may counteract inflammation and post-sepsis sarcopenia through mTOR pathway activation, suppressing hyperautophagy, a key driver of muscle atrophy. Preclinical studies support the protective role of branched-chain amino acids (BCAAs) against sepsis-induced muscle protein degradation via inhibition of autophagy signaling in skeletal muscle [141].

**Figure 2:**
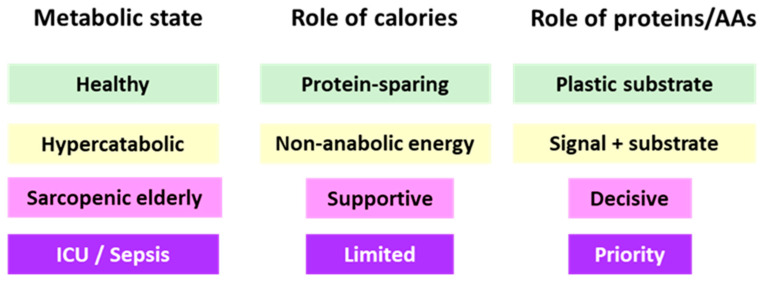
Schematic indication of the role played by calories and proteins according to metabolic state. ICU: Intensive Care Unit.

In patients with sepsis, metabolic abnormalities result in reduced muscle synthesis, resulting in decreased total muscle mass. It follows that supplementation with AAs, particularly BCAAs, plays an important role, mediated by mTOR activation, in counteracting sarcopenia following sepsis, by intervening in the continuous activation of autophagy, which is one of the causes of muscle atrophy. Although preclinical studies have shown that BCAA supplementation can prevent sepsis-induced muscle degradation by inhibiting the autophagy signaling pathway in skeletal muscles, clinical trials are still ongoing [142]. Evidence from a pilot study with 46 patients orally supplemented with a mixture of EAAs showed reduced inflammation in both inflamed and infected patients [143]. Even in the case of food supplementation, it is very important to keep in mind that AAs and their metabolites could be capable of influencing cellular metabolism and intra-organs/systems crosstalk by acting as metabokines [12]. Figure 2 schematically summarizes the different roles of calories and proteins/AAs as a function of the subject’s metabolic state.

## 9. Ultra-Processed Foods and Chronic Caloric Overfeeding: Health Implications in Critically Ill Patients

In the context of modern diets, the increasing consumption of ultra-processed foods (UPF), defined according to the NOVA classification as industrial formulations characterized by high energy density and low nutritional value, has been associated with excessive intakes of sugars and fats and with poor overall dietary quality [144]. Beyond their unfavorable macronutrient distribution, UPF are also characterized by a protein profile of low biological value compared with minimally processed foods. Indeed, many UPF rely on low-quality or additive protein sources, such as refined plant protein isolates, industrial by-products or gelatins, which often exhibit incomplete AA profiles and reduce digestibility [145]. On average, UPF provide approximately 9.5% of total energy as protein, markedly lower than the contribution observed in unprocessed or minimally processed foods (~25%), frequently leading manufacturers to supplement products with inexpensive protein ingredients of limited biological completeness [146]. Moreover, industrial processing can further impair protein digestibility and bioavailability [147].

In addition to their poor protein quality, UPF contain a high number of non-nutritive ingredients and food additives, with approximately 10,000 compounds approved for use by the FDA [148]. Collectively, these characteristics contribute to adipose tissue accumulation, metabolic dysfunction, chronic low-grade inflammation, hormonal alterations and gut microbiota dysbiosis [149]. Consequently, UPF-rich diets are consistently associated with reduced overall dietary quality and an increased risk of obesity, metabolic syndrome, type 2 diabetes and musculoskeletal dysfunctions, conditions that may also be mediated by inadequate or suboptimal protein intake [150]. These findings support the recommendation that reducing UPF consumption represents a key strategy for improving metabolic health.

Macronutrient composition plays a critical role in determining energy storage during periods of energy surplus. Experimental studies in healthy men have shown that when energy intake is increased by 50% for 14 days, excess carbohydrate intake progressively increases carbohydrate oxidation and total energy expenditure, resulting in the storage of approximately 75–85% of surplus energy. In contrast, excess fat intake minimally affects fat oxidation or energy expenditure, leading to the storage of 90–95% of excess energy, particularly during the early phase of overfeeding [151]. Consistent with these findings, UPF-rich diets in the general population are typically characterized by excessive intakes of simple sugars and saturated fats combined with inadequate protein quality and are strongly associated with increased metabolic risk [144,152]. In addition, diets high in UPF tend to displace foods rich in high-biological-value (“noble”) proteins, such as lean meats, fish, legumes and dairy products. This substitution may compromise the balance between protein synthesis and degradation, thereby facilitating the development of sarcopenia and other conditions related to metabolic aging [153].

In this context, protein emerges as a key macronutrient for optimizing body composition. Overfeeding studies indicate that dietary protein exerts protective effects against fat mass gain during energy surplus, particularly when combined with resistance training [154]. Protein intakes of approximately 1.05 g/kg/day appear sufficient to preserve lean mass under hypercaloric conditions, while intakes above 1.2 g/kg/day confer no additional benefit in sedentary individuals [155,156].

The clinical relevance of protein quality becomes even more evident in critically ill patients, in whom caloric overfeeding poses serious and potentially fatal risks. Excess energy intake relative to actual expenditure may induce hyperglycemia and insulin resistance from carbohydrate excess, hypertriglyceridemia and hepatic steatosis from excess fat or total energy intake, azotemia and metabolic acidosis from protein overfeeding in the presence of impaired nitrogen clearance, and hypercapnia due to excessive carbohydrate oxidation, thereby compromising ventilation in patients with respiratory failure [157,158,159,160]. Conversely, insufficient or poor-quality protein intake impairs protein synthesis, accelerates muscle waste and weakens immune function, all of which are critical determinants of recovery in critically ill individuals [161]. These complications are associated with prolonged ICU stays and increased mortality [160], and are exacerbated by the tendency of predictive equations to overestimate energy requirements, particularly in critically ill pediatric patients [162].

Because protein sources commonly used in UPF are characterized by incomplete AAs profiles and reduced bioavailability, these diets are typically low in EAAs, especially leucine, lysine and methionine [145]. Since EAAs cannot be synthesized endogenously, inadequate daily intake directly compromises muscle protein synthesis, favoring widespread metabolic alterations even in the presence of apparently sufficient caloric intake [68,163]. As a result, UPF-rich dietary patterns are associated with reduced EAA intake and lower overall protein quality, contributing to phenomena of “masked protein malnutrition” that may coexist with overweight and obesity [164].

In this context, supplementation with EAAs represents a potentially effective complementary nutritional strategy to counteract qualitative protein deficiencies, directly stimulate protein synthesis and support the preservation of muscle mass and metabolic function, particularly in vulnerable populations, individuals consuming UPF-rich diets or patients with increased protein requirements [165,166].

## 10. Clinical Implications and Future Perspectives

Considering the most recent scientific evidence within the framework of modern nutrition, the relationship between calories and protein warrants a substantial re-evaluation. The traditional approach, treating calories as equivalent units of energy regardless of their source, proves insufficient for accurately describing the distinct metabolic and physiological effects of different macronutrients. At an equivalent caloric intake, protein exerts effects that differ markedly from those of carbohydrates and lipids, particularly with respect to satiety, diet-induced thermogenesis, body composition, and the preservation of muscle mass. Moreover, an adequate dietary protein density and/or supplementation with all EAAs plays a critical role in regulating spontaneous energy intake and preventing the loss of lean tissue during caloric restriction. Therefore, it is necessary to move beyond a purely quantitative view of energy intake and adopt a qualitative model that incorporates metabolic function, physiological adaptations, and long-term health objectives.

In protein nutrition, it is not enough to simply consider the total amount of protein consumed; it is crucial to evaluate its AA profile, or quality. Indeed, dietary proteins differ in their content, bioavailability, and proportion of EAAs, particularly those involved in muscle protein synthesis and metabolic regulation processes. High-quality protein sources consequently provide an adequate supply of EAAs, promoting anabolic efficiency and maintaining physiological functions. Therefore, attention to protein quality and, where appropriate, EAA supplementation are key elements in nutritional support, particularly for critically ill patients.

It is necessary to move beyond a purely caloric view of nutrition and adopt a signal-based paradigm, in which nutrients (particularly EAAs) are regarded primarily as biological signals rather than mere sources of energy. Within this framework, nutrients modulate metabolic, hormonal, and epigenetic pathways that govern appetite regulation, body composition, energy metabolism, and long-term health. Consequently, the physiological impact of a food is determined not solely by its caloric content, but by its capacity to elicit specific adaptive responses within the organism. A signal-based approach to nutrition therefore provides a more robust and biologically grounded conceptual framework than simple calorie counting.

The assessment of catabolic status is not univocal and occurs through the convergence of clinical signs, active inflammation, loss of muscle mass, negative protein balance, and anabolic resistance. Although this significantly complicates clinical studies, from a practical application standpoint, it makes it imperative to assess individual metabolic profiles, as well as personalize nutritional interventions in the HCS.

It is evident that current knowledge regarding the role of proteins/AAs in the HCS is still lacking and therefore requires in-depth randomized clinical studies. It will also be necessary to investigate different nutritional supplementation strategies and consider possible pharmacological interactions.

Alterations in processes that interfere with optimal nutritional status, such as HCS, intestinal dysbiosis, and leaky gut syndrome, as well as deficiencies in molecules essential for the maintenance of anabolic pathways (e.g., AAs, vitamins and minerals), should be systematically assessed. When nutritional or metabolic components are found to be altered, targeted nutritional and therapeutic interventions should be implemented and maintained until physiological function and/or circulating concentrations are restored to normal ranges.

## 11. Limitations

This narrative review integrates mechanistic, preclinical, and clinical evidence from the nutritional and metabolic fields. While it provides a solid pathophysiological rationale for amino acid-centric nutritional strategies in the HCS, several limitations should be acknowledged.

The available evidence derives from highly heterogeneous study designs and models, including preclinical, observational, and interventional studies, with substantial variability in populations, disease contexts, endpoints, and nutritional interventions, thereby limiting comparability and generalizability. Key concepts, such as the signal-based role of EAAs and the metabokine function of AAs, are supported predominantly by animal and mechanistic studies, with incomplete clinical validation.

Moreover, existing clinical trials are often short in duration, underpowered, and methodologically diverse, precluding definitive conclusions regarding clinical efficacy, optimal dosing and long-term safety. The lack of standardized diagnostic criteria for HCS further complicates interpretation, as many studies do not adequately stratify participants by age, sex, comorbidities, inflammatory burden, insulin resistance, or concomitant pharmacological therapies. Finally, most studies do not systematically address the contribution of other nutritional factors, such as micronutrients, lipid intake, meal timing, and overall dietary patterns, nor their interactions with drug treatments, which may substantially influence metabolic outcomes.

## 12. Conclusions

Protein and AA requirements in the HCS cannot be inferred from healthy individuals, and energy provision alone is insufficient to preserve lean body mass. In these conditions, the interaction between protein and energy intake is profoundly shaped by the underlying metabolic milieu.

The HCS represents a clinically relevant phenotype shared by many acute and chronic diseases, characterized by systemic inflammation, anabolic resistance, and accelerated muscle proteolysis. As skeletal muscle functions as a major metabolic reservoir, its progressive depletion is strongly associated with adverse outcomes. Notably, in the HCS protein and AA requirements become partially uncoupled from energy needs, challenging traditional calorie-centered nutritional paradigms and supporting a shift toward “signal-based” nutrition.

This review underscores that caloric-focused strategies fail to meet the metabolic demands of hypercatabolism. Instead, growing evidence supports AA-centered interventions, particularly with EAA supplementation, which act not only as substrates but also as bioactive regulators of metabolic and signaling pathways, consistent with their role as metabokines. Marked interindividual variability in hypercatabolic responses further highlights the need for personalized, mechanism-based nutritional approaches, in which protein quality, AA composition, and timing are critical determinants of efficacy. Nevertheless, translating mechanistic insights into clinical practice remains a key challenge, emphasizing the need for well-designed clinical trials, validated metabolic biomarkers, and integrated omics strategies to better define patient-specific nutritional requirements.

## Figures and Tables

**Table 1:** Most recent review regarding protein metabolism. MPS = muscle protein synthesis; EAAs = essential amino acids; AAs = amino acids.

Study [Ref.]	Title	Main Message
Cruz-Pierard et al., 2026 [22]	Synergistic effects of protein intake and resistance exercise.	Systematic review showing protein supplementation combined with resistance exercise enhances metabolic and anabolic biomarkers related to MPS, suggesting an interplay between nutrition and physical activity.
Prokopidis et al., 2025 [23]	Food matrix in the context of muscle and whole-body protein synthesis: a scoping review.	Highlights how the food matrix (nutrient interactions within whole foods) may influence MPS and whole-body protein synthesis beyond isolated protein dose. Limited data available, calling for future trials examining whole-food context.
Coker, and Coker, 2025 [24]	Dietary proteins, AAs and insulin resistance: mini review.	Discusses how dietary protein and EAAs (especially leucine) influence insulin sensitivity and metabolic regulation, balancing anabolic stimulus with risks of chronic mTOR activation under nutrient overload.
Yimam et al., 2025 [25]	Postprandial aminoacidemia after alternative protein sources.	Focuses on postprandial AA kinetics following ingestion of alternative proteins, underlining differences in AA availability that can modulate metabolic responses and protein turnover.
Matthews et al., 2025 [26]	Understanding dietary protein quality.	Reviews methods to assess dietary protein quality emphasize how quality metrics relate to digestibility, EAAs content, and metabolic impacts including protein synthesis stimulation.

**Table 2:** Effect of total caloric intake combined with protein intake on maintenance of lean body mass (LBM) in hypercatabolic states. E.E. = Energy Expenditure; AAs = Amino Acids. Arrow down = decrease; Arrow up = increase.

Nutritional Strategy	Total Caloric Intake % E.E.	Protein Intake g/kg/day	Effect on LBM	Predominant Mechanisms	[Ref.]
Very low calories + low protein	<50%	<1.0	Rapid loss	↑ gluconeogenesis from AA, ↑ proteolysis, ↓ protein synthesis	[64]
Low calories + high protein	<60–70%	≥1.5–2.0	Partial preservation	AAs oxidation for energy purposes, incomplete protein-sparing effect	[61,62]
Adequate calories + adequate protein	70–100%	1.2–1.5	Better preservation	↓ AAs oxidation, ↓ gluconeogenesis, insulin effect	[91,93]
Adequate calories + high protein	70–100%	1.5–2.0	Maximum possible preservation (Stable phase)	↓ proteolysis, ↑ protein synthesis (Limited by anabolic resistance)	[61,94]
High calories + Very high protein	>110–120%	≥2.0	No additional benefits	Overfeeding, lipogenesis, ↑ metabolic stress	[95,96]

**Table 3:** Summary of studies evaluating the effects of total energy and protein intake on lean body mass (LBM) preservation in HCS. Overall, despite substantial heterogeneity among studies, protein intake above a defined threshold does not appear to confer additional benefits for LBM, whereas inadequate total energy intake is consistently associated with impaired LBM maintenance or loss. RE = resistance exercise. ICU = intensive care unit.

Study [Ref.]	Population	Study Design	Energy Intake	Protein Intake g/kg/day	Comparator	Outcome on LBM	Key Findings
Stein et al., 2024. [99]	Obese	Additional protein intake in preservation of LBM	Hypo	1.5	Lower intake (1 g/kg/day)	significantly reduced in both groups	no differences in weight loss between the groups
Nunes et al., 2022 [100]	healthy adult (18 years or older)	Randomized controlled trial	Normo	1.2–1.59 and >1.6	Placebo or no intervention	increasing daily protein ingestion may enhance gains in LBM in studies enrolling subjects in RE	increasing protein ingestion results in small additional gains in LBM and lower body muscle strength gains.
Arends et al., 2017 [101]	Cancer patients	Clinical guidelines (ESPEN)	Normo/ Hyper	1.2–1.5	Inadequate intake	Partial preservation of LBM	Energy adequacy required to overcome anabolic resistance
Longland et al., 2016 [102]	Young man	Single-blind, randomized, parallel-group trial. RE training with high-intensity interval training.	Hypo	2.4	Lower intake (1.2 g/kg/day)	LBM increased in the higher protein group and loss of fat mass.	High protein diet was more effective in promoting increases in LBM and losses of fat mass when combined with high intensity RE and anaerobic exercise.
Weijs et al., 2014 [93]	ICU patients	Observational cohort	≥80% measured energy expenditure	≥1.2	Lower intake	Reduced mortality and muscle loss	Best outcomes when energy and protein targets met together
Casaer et al., 2011 [95]	ICU patients	Randomized controlled trial	Early vs. late parenteral nutrition	~1.2	Early high-calorie PN	Less muscle waste with delayed calories	Early full calories blunt benefits of protein in acute phase
Villet et al., 2005 [103]	ICU patients	Prospective observational study	Hypo	~1.0	Adequate energy/protein	Progressive LBM loss	Energy deficit strongly associated with loss of fat-free mass
Paddon-Jones et al., 2004 [104]	Healthy adults (catabolic model)	Controlled feeding trial	Normo	High-quality protein + EAAs	Lower protein	Improved muscle protein synthesis	Adequate energy enhances anabolic response to protein
Wolfe et al., 2000 [105]	Critically ill patients	Narrative review of metabolic studies	Iso/ Hyper	≥1.5	Hypocaloric, low protein	Partial preservation of LBM	Energy–protein synergy limits endogenous protein oxidation
Wolfe et al., 1983 [106]	Severe burn patients	Metabolic balance study	Hyper	2.0–2.5	Lower protein intake	Improved nitrogen balance; reduced LBM loss	Adequate calories are required for protein to exert anabolic effects

**Table 4:** More recent systematic reviews of RCTs on effects of protein supplementation in critical illness. SR = systematic review; MA = meta-analysis; RCT = randomized clinical trial; ICU = intensive care unit.

Study [Ref.]	Study Design	Study Objective	Main Findings
Hu et al., 2025 [107]	SR + MA	To evaluate the association between different levels of protein intake and renal adverse events and mortality in critically ill patients	Protein intake >1.3 g/kg/day was not associated with an increased risk of renal adverse events; no significant differences in 28-day, ICU, or hospital mortality were observed
Castro et al., 2025 [108]	SR + MA	To assess the impact of different protein intakes in patients with chronic critical illness	Higher protein intake (>1.3 g/kg/day) was associated with reduced early mortality, with no effect on late mortality or other clinical outcomes
Badpeyma et al., 2025 [109]	MA	Evaluating dose–response between high vs. low protein in ICU patients	No significant effect on mortality or length of stay; reduction in muscle atrophy with high protein intake
Badpeyma et al., 2025 [110]	RCT	Comparing high protein (2.2 g/kg/day) vs. conventional (1.0 g/kg/day) on mortality and clinical outcomes	High protein dose showed no clear improvement in mortality or major outcomes, highlighting uncertainties about efficacy and safety
Mohamed et al., 2025 [94]	SR+MA	To systematically determine the effect of protein dose (high >2.2 g/kg/day vs. low <1.2 g/kg/day) on skeletal muscle strength in critically ill patients	Significant difference in skeletal muscle strength with higher vs. lower protein intakes. Inconsistency was evident across the included studies.
O’Keefe et al., 2025 [111]	RCT	To test the hypothesis that supplementing enteral protein intake (2 g/kg/day vs. standard nutritional care) would improve outcomes.	Protein supplementation did not improve outcomes but was associated with increased complications
Wang et al., 2024 [112]	RCT	To evaluate whether early high protein intake (1.5 g/kg/day) improves prognosis compared with low intake (0.8 g/kg/day)	No significant reduction in 28-day mortality; potential favorable effects on muscle preservation and duration of mechanical ventilation were reported
Qin et al., 2024 [113]	SR + MA	To compare higher (≥1.2 g/kg/day) vs. lower (<1.2 g/kg/day) protein intake on clinical outcomes in adult ICU patients	No significant differences were found in mortality, ICU or hospital length of stay, duration of mechanical ventilation, or incidence of acute kidney injury
Blaauw et al., 2024 [114]	SR	To compare a protein intake group (≥1.2 g/kg/day) with a lower protein intake group (<1.2 g/kg/day) in critically ill adult patients on mortality, length of ICU and hospital stay.	Higher protein group results in little to no difference in mortality, with a probable slight increase in length of ICU stay and length of hospital stay.
Bels et al., 2023 [62]	SR	Protein supplementation in ICU patients.	Protein supplementation has shown positive effects on recovery and mortality.

## Data Availability

No new data were created or analyzed in this study. Data sharing is not applicable to this article.

## Abbreviations

The following abbreviations are used in this manuscript:

AAs	Amino acids
CEAAs	Conditionally essential amino acids
DIAAS	Digestible indispensable amino acid score
EAAs	Essential amino acids
eNOS	Endothelial nitric oxide synthase
GLP-1	Glucagon-like peptide-1
HCS	Hyper catabolic state
ICU	Intensive care unit
LBM	Lean body mass
MPS	Muscle protein synthesis
mTORC1	Mechanistic target of rapamycin complex-1
NB	Nitrogen balance
NEAAs	Non-essential amino acids
PSMF	protein-sparing modified fast
TLR	Toll-like receptors
UPS	Ultra-processed foods
